# Systemic Immune-Inflammation Index as a Prognostic Marker in Gastric and Gastroesophageal Junction Cancers Receiving Perioperative FLOT Therapy

**DOI:** 10.3390/medicina61091614

**Published:** 2025-09-06

**Authors:** Pinar Peker, Asli Geçgel, Oğuzcan Özkan, Ivo Gökmen, Serkan Menekse, Alpay Duşgun, Berna Bozkurt Duman, Timuçin Çil

**Affiliations:** 1 Department of Medical Oncology, Adana City Training and Research Hospital, Adana 01370, Turkey; alpaydusgun@hotmail.com (A.D.); berboz@hotmail.com (B.B.D.); drtimucincil@gmail.com (T.Ç.); 2 Department of Medical Oncology, Faculty of Medicine, Ege University, Izmir 35100, Turkey; dr.asltrgt@gmail.com (A.G.); droguzcanozkan@yahoo.com (O.Ö.); 3 Department of Medical Oncology, Mehmet Akif Ersoy Public Hospital, Çanakkale 17100, Turkey; ivo_georgiev1@hotmail.com; 4 Department of Medical Oncology, Manisa City Hospital, Manisa 45040, Turkey; drsermen@hotmail.com

**Keywords:** systemic immune-inflammation index (SII), gastric cancer, gastroesophageal junction cancer, FLOT chemotherapy, prognostic biomarker

## Abstract

*Background and Objectives*: The systemic immune-inflammation index (SII), derived from peripheral blood parameters, has emerged as a novel marker reflecting the balance between host immunity and tumor-related inflammatory burden. This study aimed to investigate the prognostic impact of baseline SII on survival outcomes in patients with gastric or gastroesophageal junction (GEJ) cancer undergoing perioperative FLOT (5-fluorouracil, leucovorin, oxaliplatin, and docetaxel) chemotherapy. *Materials and Methods*: In this retrospective study, 168 patients with histologically confirmed gastric or GEJ cancer who received perioperative FLOT therapy were included. SII was calculated using the formula: SII = (Platelets × Neutrophils)/Lymphocytes. ROC curve analysis determined the optimal SII cutoff for predicting mortality. Patients were categorized into low (SII ≤685) and high (SII >685) groups. Overall survival (OS) and disease-free survival (DFS) were evaluated using Kaplan–Meier analysis and compared via the log-rank test. Cox proportional hazards regression models were used for univariate and multivariate analysis. *Results*: The optimal cutoff value for SII was determined to be 685 (AUC: 0.652, 95% CI: 0.558–0.747, *p* = 0.003). High SII was significantly shorter OS (17.4 vs. 28.2 months, *p* = 0.001) Multivariate analysis identified high SII (HR = 1.88, 95% CI: 1.36–2.89, p = 0.039), advanced T stage (HR = 3.693, *p* < 0.001), poor treatment response (HR = 0.36, *p* < 0.001), and ECOG-PS ≥1 (HR = 3.297, *p* < 0.001) as independent predictors of mortality. *Conclusions*: Elevated baseline SII is an independent predictor of worse OS and DFS in gastric and GEJ cancer patients receiving perioperative FLOT chemotherapy. SII may serve as a practical and inexpensive biomarker to support risk stratification and personalized treatment decisions.

## 1. Introduction

As the fourth most prevalent cause of cancer-related deaths globally and the fifth most often diagnosed cancer, gastric cancer (GC) and GEJ cancer continue to represent major global health burdens, according to GLOBOCAN 2020 estimates [1]. Current treatment strategies for locally advanced gastric and GEJ cancers include surgery, chemotherapy, radiotherapy, targeted therapy, and immunotherapy [2]. Despite advancements in diagnostic modalities and the implementation of multimodal treatment approaches (particularly perioperative chemotherapy regimens such as FLOT), overall survival (OS) in these patients remains suboptimal due to high recurrence rates and limited long-term disease control [3].

Systemic inflammation plays a crucial role in cancer development and progression by promoting tumor growth, angiogenesis, metastasis, immune evasion, and therapy resistance. Key inflammatory mediators within the tumor microenvironment include cytokines such as macrophage migration inhibitory factor (MIF), tumor necrosis factor-alpha (TNF-α), and interleukin-6 (IL-6) [4,5]. Among the immune cells within the tumor inflammatory microenvironment, neutrophils and platelets support tumor growth and immune evasion, whereas lymphocytes play a protective role. The balance among these cell types can be easily assessed through routine complete blood count tests and may reflect the host’s underlying immune and inflammatory status [6].

The SII, calculated using the formula platelet count × neutrophil count/lymphocyte count, has emerged as a promising and accessible biomarker that integrates these three fundamental hematological parameters [7]. Based on peripheral blood neutrophil, platelet, and lymphocyte levels, SII reflects various inflammatory and immunological pathways in vivo and is considered a highly stable and easily calculable inflammatory index [8]. Numerous studies have demonstrated the prognostic value of SII in various malignancies, including hepatocellular carcinoma, non-small cell lung cancer, and colorectal cancer [9,10,11,12]. Elevated SII levels have been consistently associated with poor OS, increased recurrence rates, and more aggressive tumor phenotypes.

However, the prognostic value of SII in gastric and GEJ cancers, particularly in patients receiving perioperative chemotherapy, remains inadequately defined. Since the inflammatory response within the tumor microenvironment can influence both treatment response and survival, evaluating biomarkers such as SII prior to therapy is of great importance [13]. Although some retrospective studies have suggested an association between elevated SII levels and poor prognosis in GC [14], data regarding its predictive performance specifically in patients receiving perioperative treatment with the FLOT regimen remain limited. The association between the tumor inflammatory microenvironment and the development and spread of GC underscores the importance of understanding the immune response within the tumor microenvironment, as this response is known to play a critical role in tumor progression and metastasis. Targeting inflammatory pathways may offer novel therapeutic strategies to improve treatment outcomes in patients with GC [15].

In this study, we aimed to investigate the prognostic impact of baseline SII on survival outcomes in patients with histologically confirmed gastric or GEJ cancers undergoing perioperative FLOT chemotherapy. We hypothesized that higher baseline SII levels would be associated with poorer clinical outcomes and could serve as a simple, cost effective biomarker for risk stratification.

## 2. Materials and Methods

### 2.1. Study Design and Patient Selection

This retrospective cohort study included 168 patients diagnosed with histologically confirmed gastric or GEJ cancer who received perioperative chemotherapy with the FLOT protocol (5-fluorouracil, leucovorin, oxaliplatin, and docetaxel) between January 2019 and March 2025. Patients were identified through the institutional oncology database.

The inclusion criteria were as follows: age ≥18 years, histologically confirmed gastric or GEJ cancers, availability of complete pre-treatment hematologic and clinical data, receipt of at least one cycle of perioperative FLOT chemotherapy, and availability of follow-up data, including treatment response, recurrence, and survival outcomes. Exclusion criteria were: presence of active infection, autoimmune disease, or chronic inflammatory condition at diagnosis, history of another synchronous malignancy, and incomplete clinical or laboratory data.

### 2.2. Data Collection

Clinical and demographic data were retrieved from electronic medical records. Variables included age, sex, body mass index (BMI), smoking history, blood group, Eastern Cooperative Oncology Group Performance Score (ECOG-PS), tumor location (proximal or distal), histologic subtype, clinical and pathological TNM stage, presence of perineural invasion (PNI) and lymphovascular invasion (LVI), surgical resection status, treatment response, recurrence, and survival. OS was defined as time from diagnosis to death from any cause or last follow-up. DFS was defined as time from curative surgery to documented recurrence or death.

Hematologic parameters (neutrophil, lymphocyte, and platelet counts) were obtained from complete blood count tests performed within one week before the initiation of chemotherapy using an automated hematology analyzer (Sysmex XN-1000, Sysmex Corporation, Kobe, Japan). The SII was calculated using the following formula: SII = [neutrophil (cells × 10^9^/L) × platelet (cells × 10^9^/L)]/lymphocyte (cells × 10^9^/L).

### 2.3. SII Cutoff Determination

Receiver operating characteristic (ROC) curve analysis was performed to determine the optimal SII cutoff for predicting overall mortality. The cutoff maximizing sensitivity and specificity was selected, resulting in a threshold value of 685. Patients were classified into low (≤685) and high (>685) SII groups accordingly.

### 2.4. Statistical Analysis

All statistical analyses were performed using IBM SPSS Statistics version 25.0 (IBM Corp., Armonk, NY, USA). Descriptive statistics were reported as medians and interquartile ranges (IQRs) for continuous variables, and as frequencies and percentages for categorical variables. Comparisons between low and high SII groups were made using Pearson’s chi-square test or Fisher’s exact test, as appropriate. Survival analyses were conducted using the Kaplan–Meier method, and differences between groups were assessed using the log-rank test.

Univariate Cox proportional hazards regression was used to identify variables associated with overall mortality. Variables with a *p*-value < 0.1 in the univariable Cox regression were entered into the multivariable Cox regression model. Variables that lost statistical significance in the multivariable analysis are reported as not significant (NS). Although both OS and DFS were evaluated, due to the relatively short follow-up and overlapping recurrence and mortality events, DFS results were presented only graphically, and OS was prioritized in tabular analyses to maintain clarity.

## 3. Results

### 3.1. Baseline Characteristics

A total of 168 patients with histologically confirmed gastric or GEJ cancers were included in the study. Patient demographics and tumor characteristics are detailed in Table 1. The median age at diagnosis was 64 years (range: 33–83), and the majority of patients were male (62.5%). Most tumors were located distally (70.8%), with intestinal adenocarcinoma being the predominant histologic subtype (67.9%). The lifestyle and comorbidity status of the patients revealed that 61.3% were smokers, and 39.9% had at least one chronic comorbidity. The median BMI was 24.7 kg/m^2^, and patients were roughly evenly divided between a BMI < 25 and >25 kg/m^2^.

The majority of patients had favorable baseline Eastern Cooperative Oncology Group performance status (ECOG-PS) from a hematologic and clinical standpoint; 79.2% of patients presented with ECOG 0 and 20.8% with ECOG ≥1. With one quarter of patients having blood group O (25.6%) and three quarters not having blood group O (74.4%), the distribution of blood groups was in line with that of the general population.

With respect to tumor features, distal gastric tumors were predominant (70.8%) compared with proximal/GEJ cancers (29.2%). Histologically, the intestinal-type adenocarcinoma was more frequent (67.9%), whereas signet-ring cell carcinoma accounted for 32.1% of cases. In terms of pathologic staging, the majority of patients had advanced primary tumors (T3–4: 63.8%) and nodal involvement (N+: 63.1%). Perineural invasion (PNI) and lymphovascular invasion (LVI) were also common, observed in 73.2% and 66.7% of patients, respectively.

### 3.2. Prognostic Value of SII: ROC Analysis

The discriminatory ability of baseline SII in predicting overall mortality was evaluated using receiver operating characteristic (ROC) curve analysis. According to the analysis, SII demonstrated a moderate discriminatory ability, with an area under the curve (AUC) of 0.652 (95% CI: 0.558–0.747; *p* = 0.003), indicating limited but statistically significant prognostic value.

**Figure 1:**
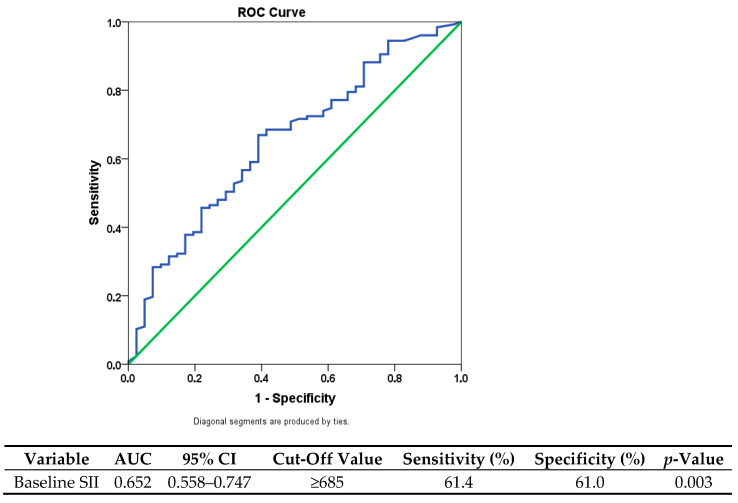
Receiver operating characteristic (ROC) curve for SII predicting overall survival. The blue line represents the ROC curve, and the green diagonal line indicates the reference line (AUC = 0.5).

The ideal cutoff point was found to be 685, which offered a balanced threshold for clinical use with a sensitivity of 61.4% and a specificity of 61.0%. Patients were divided into two groups based on this value: those with low SII (≤685; *n* = 74) and those with high SII (>685; *n* = 94). This classification had significant clinical ramifications in addition to making statistical comparisons easier. While patients in the high-SII group showed signs of aggressive tumor biology and a higher chance of an unfavorable prognosis, those in the low-SII group typically showed better disease characteristics and survival outcomes (Figure 1).

### 3.3. Univariate and Multivariate Cox Regression Analysis

Univariate Cox regression analysis identified several clinicopathological variables that significantly influenced overall survival. Adverse prognostic factors included low body mass index (<25), distal tumor location, absence of surgical resection, advanced tumor stage (T3–T4), nodal metastasis, perineural invasion (PNI), lymphovascular invasion (LVI), impaired performance status (ECOG-PS ≥1), and elevated SII (>685). Conversely, patients with a higher BMI (≥25) or those who underwent curative surgery demonstrated improved outcomes, consistent with the survival advantage conferred by adequate nutritional status and effective local disease control.

When these variables were entered into the multivariate model, several retained their independent prognostic significance. Advanced tumor stage (HR = 3.69; 95% CI: 2.07–6.58; *p* < 0.001), impaired performance status (ECOG-PS ≥1; HR = 3.30; 95% CI: 1.74–6.25; *p* < 0.001), elevated baseline SII (>685; HR = 1.88; 95% CI: 1.36–2.89; *p* = 0.039), and poor or absent response to neoadjuvant therapy (HR = 0.36; 95% CI: 0.21–0.61; *p* < 0.001 for complete responders) emerged as independent predictors of mortality. These findings underscore the interplay between host-related factors (such as systemic inflammation and functional status), tumor burden, and treatment response in shaping survival outcomes.

A summary of the univariate and multivariate Cox regression analysis is provided in Table 2.

### 3.4. Survival Analysis

Kaplan–Meier survival analysis confirmed the association between elevated baseline SII and poorer survival outcomes. The median OS for patients with SII ≤685 was 28.2 months, whereas it was 17.4 months for those with SII >685, and this difference was statistically significant (log-rank *p* = 0.001). In the subgroup survival analysis by age (≤65 vs. >65 years), no statistically significant difference was observed between the two groups in terms of OS and DFS. The median OS was 13.1 months (95% CI: 10.53–15.67) in the ≤65 years group and 11.0 months (95% CI: 8.88–13.12) in the >65 years group, with no significant difference (*p* = 0.721). Similarly, DFS results did not differ significantly between the age groups. These findings indicate that, in our cohort, age alone was not an effective prognostic factor.

**Figure 2:**
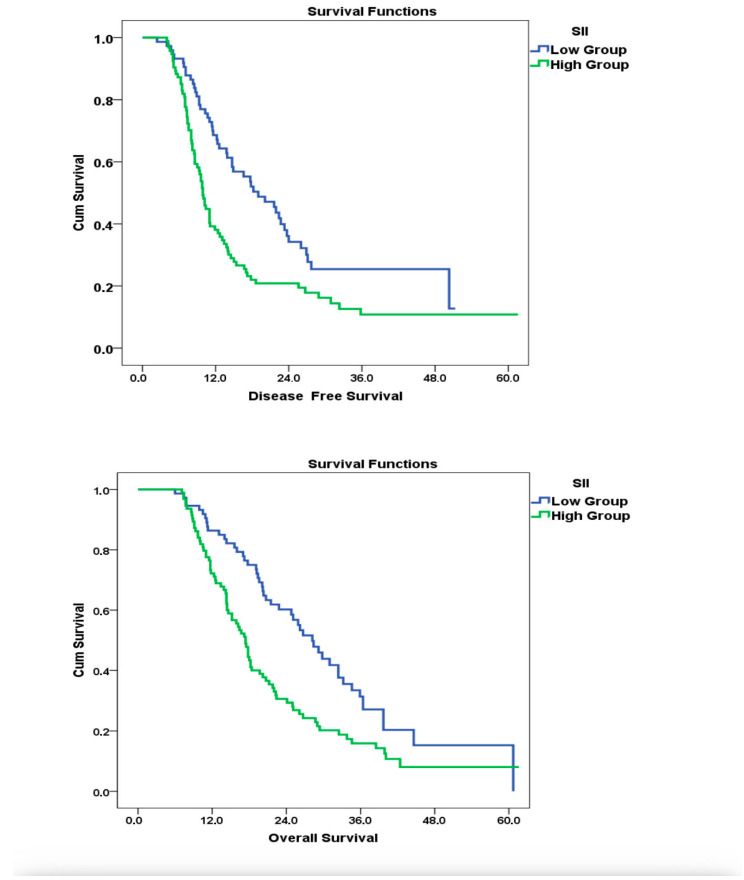
Impact of SII at Diagnosis on OS and DFS: Kaplan–Meier and Cox Regression Analysis.

Consistently, in the multivariate Cox regression analysis, high SII values were independently associated with increased mortality risk (1.88, 95% CI: **1.36–2.89**, *p* = 0.039), supporting the prognostic relevance of systemic inflammation. The adverse impact of elevated SII on DFS was also evident, with significantly shorter median DFS observed in the high SII group (9.9 vs. 19.0 months; *p* = 0.001). Kaplan–Meier survival curves and corresponding Cox regression models illustrating these findings are presented in Figure 2. However, consistent with our predefined analysis plan, DFS outcomes were displayed graphically (Figure 2) but not included in the main regression tables due to limited follow-up.

## 4. Discussion

This study demonstrates that the SII is an independent and clinically relevant prognostic biomarker in patients with gastric and GEJ cancer receiving perioperative FLOT chemotherapy. Elevated baseline SII levels were associated with inferior OS, regardless of treatment response and tumor stage. To our knowledge, this is among the first studies to evaluate the prognostic value of SII in this specific treatment setting, supporting the emerging role of inflammatory biomarkers in oncologic risk stratification and clinical decision-making.

In our study, the optimal SII cutoff value was 685, which is higher than the values reported in other gastric cancer cohorts (395–892) [16,17,18]. This discrepancy may reflect differences in patient populations, treatment regimens, and timing of blood sampling.

Importantly, this cutoff requires external validation before clinical application, as highlighted in recent systematic reviews and meta-analyses [19,20]. Although the discriminatory ability in our cohort was moderate (AUC = 0.652), elevated SII consistently correlated with worse survival outcomes across studies and may outperform other inflammation-based indices such as NLR and PLR. Our findings align with previous studies showing that high SII levels (>685) are associated with shorter OS and DFS in gastric and GEJ cancers. In our cohort, patients with elevated systemic inflammation—marked by increased neutrophils and platelets and reduced lymphocytes—more often had advanced tumor stage (T3–T4: 63.8%), nodal metastases (63.1%), and received perioperative FLOT chemotherapy. Future studies incorporating molecular profiling and cytokine analyses may help clarify the biological mechanisms underlying these variations.

Although many studies have investigated the prognostic value of SII in gastric cancer, results have been inconsistent across individual cohorts. Some analyses reported limited significance compared with other markers such as NLR or PLR [19], whereas meta-analyses consistently confirmed that elevated pre-treatment SII is associated with poorer survival outcomes, independent of treatment strategy or cutoff values [20]. Unlike some prior studies suggesting age-dependent differences in the prognostic value of SII [21,22,23], our cohort showed no significant impact of age on OS or DFS.

The literature also indicates that high SII levels are associated with advanced TNM stage and greater tumor invasion depth [24], its prognostic relevance may be particularly pronounced in subtypes such as signet-ring cell gastric cancer, and it has also been compared with other indices such as NLR, PLR, or MLR [25,26].

In our study, patients with a BMI ≥ 25 initially showed better survival in univariate analysis, suggesting a possible “obesity paradox.” However, this association was not confirmed in the multivariate model, indicating that it should be interpreted only as an exploratory finding rather than a robust prognostic factor. Similar paradoxical associations have been reported particularly in lung cancer, renal cell carcinoma, and some gastrointestinal cancers [27,28,29]. Moreover, mechanistic explanations have been suggested, as increased energy reserves and better nutritional status in obese individuals may enhance tolerance to chemotherapy and other systemic treatments, potentially providing a survival advantage [30,31,32].

Other biomarkers such as fibrinogen or TLS have also been linked to prognosis in gastric cancer [33,34], but these were beyond the scope of our analysis. Building on this evidence, our study evaluates the prognostic value of SII in gastric and GEJ cancer patients treated with the modern perioperative FLOT regimen, thereby contributing to the understanding of its clinical relevance.

Beyond survival outcomes, elevated SII was strongly associated with adverse pathological features. Several studies have demonstrated its link with lymphovascular invasion and perineural invasion [35,36]. Additional reports have further confirmed that high SII increases the prognostic significance of these factors in gastric and other malignancies [37,38,39]. These associations support the concept that SII reflects aggressive tumor biology. Importantly, this simple and inexpensive biomarker may complement conventional TNM staging and enhance risk stratification across different clinical settings. Given these biological associations, it is important to acknowledge several limitations of our study. First, as a single-center retrospective analysis, potential selection bias may limit the generalizability of our findings. Second, the lack of external validation and absence of molecular or genomic data (e.g., MSI status, HER2 expression, PD-L1 levels) restricts a more comprehensive understanding of the role of systemic inflammation in tumor biology. Third, the optimal SII cutoff derived from our cohort may not be universally applicable. Fourth, although both OS and DFS were recorded, DFS results were not presented in the main tables due to the short follow-up period, which led to substantial overlap between recurrence and mortality events; thus, OS was prioritized to maintain analytical clarity. Therefore, DFS findings were restricted to graphical representation only, and OS remained the primary endpoint for tabular reporting. Finally, no dedicated subgroup analysis was performed for the partial response group, as it did not demonstrate independent prognostic significance in the multivariate model. Nevertheless, the prognostic relevance of systemic inflammation in this subgroup remains uncertain and warrants investigation in larger, prospectively designed studies. Despite these limitations, our study has several notable strengths. The cohort was relatively large for a single-center analysis and consisted exclusively of patients receiving perioperative FLOT according to standardized protocols, which minimized treatment-related heterogeneity. Comprehensive clinicopathologic characterization and complete follow-up data enhanced the reliability of survival analyses. Furthermore, SII was calculated from routinely available hematological parameters, supporting its feasibility and potential applicability in diverse clinical settings, including those with limited resources. Collectively, these strengths reinforce the validity of our findings and their relevance to real-world practice.

In summary, our findings indicate that elevated SII not only mirrors systemic inflammation but also reflects tumor aggressiveness. This underscores its potential utility as a simple and robust biomarker for clinical risk stratification.

## 5. Conclusions

In conclusion, elevated baseline SII is an independent predictor of poor survival in patients with gastric and GEJ cancers undergoing perioperative FLOT chemotherapy and is significantly associated with adverse clinicopathological features, poor treatment response, and markedly worse OS and DFS. While our study provides compelling evidence on its prognostic utility, further large-scale prospective multicenter studies with external validation and molecular correlation are warranted to confirm these results, clarify its predictive role for treatment response, and refine its integration into personalized treatment strategies.

## Figures and Tables

**Table 1:** Baseline Demographic and Clinical Characteristics of the Patients.

Variable	Total (*n* = 168)	SII ≤ 685 (*n* = 74)	SII > 685 (*n* = 94)	*p*-Value
Age group	≤65: 87 (51.8%) >65: 81 (48.2%)	≤65: 34 (45.9%) >65: 40 (54.1%)	≤65: 53 (56.4%) >65: 41 (43.6%)	0.179
Sex	Male: 105 (62.5%) Female: 63 (37.5%)	Male: 50 (67.6%) Female: 24 (32.4%)	Male: 55 (58.5%) Female: 39 (41.5%)	0.229
Blood group (O/Non-O)	O: 43 (25.6%) Non-O: 125 (74.4%)	O: 22 (29.7%) Non-O: 52 (70.3%)	O: 21 (22.3%) Non-O: 73 (77.7%)	0.276
Smoking history	Yes: 103 (61.3%) No: 65 (38.7%)	Yes: 47 (63.5%) No: 27 (36.5%)	Yes: 56 (59.6%) No: 38 (40.4%)	0.603
Comorbidity	Yes: 67 (39.9%) No: 101 (60.1%)	Yes: 30 (40.5%) No: 44 (59.5%)	Yes: 37 (39.4%) No: 57 (60.6%)	0.877
BMI (<25/≥25)	<25: 93 (55.4%) ≥25: 75 (44.6%)	<25: 41 (55.4%) ≥25: 33 (44.6%)	<25: 52 (55.3%) ≥25: 42 (44.7%)	0.991
Tumor histology	Adenocarcinoma: 114 (67.9%) Signet-ring cell carcinoma: 54 (32.1%)	Adenocarcinoma: 51 (68.9%) Signet-ring cell carcinoma: 23 (31.1%)	Adenocarcinoma: 63 (67.0%) Signet-ring cell carcinoma:31 (33.0%)	0.794
Tumor location	Proximal: 49 (29.2%) Distal: 119 (70.8%)	Proximal: 18 (24.3%) Distal: 56 (75.7%)	Proximal: 31 (33.0%) Distal: 63 (67.0%)	0.221
Surgical resection	Yes: 123 (73.2%) No: 45 (26.8%)	Yes: 58 (78.4%) No: 16 (21.6%)	Yes: 65 (69.1%) No: 29 (30.9%)	0.180
T stage *	T1–2: 51 (36.2%) T3–4: 90 (63.8%)	T1–2: 31 (47.0%) T3–4: 35 (53.0%)	T1–2: 20 (26.7%) T3–4: 55 (73.3%)	0.012
N stage *	N0: 52 (36.9%) N+: 89 (63.1%)	N0: 33 (50.0%) N+: 33 (50.0%)	N0: 19 (25.3%) N+: 56 (74.7%)	0.002
Neoadjuvant response	No response: 70 (41.7%) Partial: 56 (33.3%) Complete: 42 (25.0%)	No response: 21 (28.4%) Partial: 25 (33.8%) Complete: 28 (37.8%)	No response: 49 (52.1%) Partial: 31 (33.0%) Complete: 14 (14.9%)	0.001
PNI	Present: 123 (73.2%) Absent: 45 (26.8%)	Present: 51 (68.9%) Absent: 23 (31.1%)	Present: 72 (76.6%) Absent: 22 (23.4%)	0.265
LVI	Present: 112 (66.7%) Absent: 56 (33.3%)	Present: 39 (52.7%) Absent: 35 (47.3%)	Present: 73 (77.7%) Absent: 21 (22.3%)	0.001
ECOG-PS	ECOG 0: 133 (79.2%) ECOG ≥1: 35 (20.8%)	ECOG 0: 64 (86.5%) ECOG ≥1: 10 (13.5%)	ECOG 0: 69 (73.4%) ECOG ≥1: 25 (26.6%)	0.038
Recurrence	Yes: 135 (80.4%) No: 33 (19.6%)	Yes: 53 (71.6%) No: 21 (28.4%)	Yes: 82 (87.2%) No: 12 (12.8%)	0.011
Mortality	Deceased: 127 (75.6%) Alive: 41 (24.4%)	Deceased: 49 (66.2%) Alive: 25 (33.8%)	Deceased: 78 (83.0%) Alive: 16 (17.0%)	0.012

* T and N staging available for SII ≤ 685 group: *n* = 66; SII > 685 group: *n* = 75. **Abbreviations:** SII—Systemic Immune-Inflammation Index; BMI—Body Mass Index; NOS—Not Otherwise Specified; PNI—Perineural Invasion; LVI—Lymphovascular Invasion; ECOG—Eastern Cooperative Oncology Group.

**Table 2:** Univariate and Multivariate Cox Regression Analysis.

Variable	Median OS (95% CI)	Univariable HR (95% CI)	*p*-Value	Multivariable HR (95% CI)	*p*-Value	Reference Category
Age > 65	11.0 (8.88–13.12)	1.06 (0.76–1.49)	0.721	–	–	≤65 years
Male sex	12.7 (9.97–15.43)	0.84 (0.60–1.20)	0.340	–	–	Female
Non-0 blood group	11.6 (10.06–13.14)	1.21 (0.81–1.81)	0.345	–	–	Blood group 0
Smoking	11.5 (9.38–13.62)	1.04 (0.73–1.46)	0.847	–	–	Non-smoker
Comorbidity present	11.0 (8.87–13.13)	0.95 (0.67–1.35)	0.783	–	–	None
**BMI ≥ 25**	16.7 (12.02–21.38)	0.60 (0.43–0.85)	0.004	0.79 (0.55–1.15)	0.220 (NS)	BMI < 25
Signet-ring cell carcinoma	11.0 (8.00–14.00)	1.30 (0.91–1.85)	0.146	–	–	NOS
**Distal tumor**	13.1 (10.87–15.33)	0.69 (0.48–0.99)	0.041	0.84 (0.55–1.28)	0.410 (NS)	GEJ/Cardia
**Surgery performed**	15.0 (12.28–17.72)	0.32 (0.22–0.47)	<0.001	0.71 (0.44–1.15)	0.162 (NS)	No surgery
**T stage (T3–4)**	11.0 (9.70–12.30)	5.74 (3.50–9.41)	<0.001	**3.69 (2.07–6.58)**	<0.001	T1–2
**N stage (N+)**	10.4 (9.38–11.42)	5.09 (3.21–8.07)	<0.001	1.44 (0.82–2.53)	0.200 (NS)	N0
**PNI present**	10.0 (8.91–11.09)	3.22 (2.06–5.05)	<0.001	1.32 (0.71–2.45)	0.380 (NS)	PNI absent
**LVI present**	9.8 (8.76–10.84)	4.04 (2.64–6.18)	<0.001	1.56 (0.84–2.87)	0.160 (NS)	LVI absent
**ECOG ≥ 1**	6.8 (6.32–7.28)	8.93 (5.67–14.06)	<0.001	**3.30 (1.74–6.25)**	<0.001	ECOG 0
**SII > 685**	9.8 (8.96–10.64)	1.82 (1.28–2.57)	0.001	**1.88 (1.36–2.89)**	0.039	SII ≤ 685
**Neoadjuvant—partial response**	14.0 (11.56–16.45)	0.18 (0.12–0.27)	<0.001	0.91 (0.53–1.55)	0.720 (NS)	No response
**Neoadjuvant—complete response**	28.9 (24.00–33.80)	0.07 (0.04–0.11)	<0.001	**0.36 (0.21–0.61)**	<0.001	No response

SII: Systemic Immune-Inflammation Index, PNI: Perineural Invasion, LVI: Lymphovascular Invasion, GEJ: Gastroesophageal Junction **Statistical Notes**: Kaplan–Meier *p*-values derived from log-rank test. Univariable and multivariable HRs calculated using Cox proportional hazards regression. NS: Not Significant. Variables without multivariable HRs were not retained in final multivariable model due to non-significance or collinearity. Variables with *p* < 0.1 in univariate analysis were entered into the multivariate model; variables not retained or losing statistical significance are indicated as NS.

## Data Availability

The original contributions presented in this study are included in the article. Further inquiries can be directed to the corresponding author.

## Abbreviations

SII:	Systemic Immune-Inflammation Index
GC:	Gastric Cancer
GEJ:	Gastroesophageal Junction
FLOT:	5-Fluorouracil, Leucovorin, Oxaliplatin, Docetaxel
OS:	Overall Survival
DFS:	Disease-Free Survival
ROC:	Receiver Operating Characteristic
AUC:	Area Under the Curve
HR:	Hazard Ratio
OR:	Odds Ratio
CI:	Confidence Interval
ECOG- PS:	Eastern Cooperative Oncology Group Performance Score
TNM:	Tumor-Node-Metastasis
PNI:	Perineural Invasion
LVI:	Lymphovascular Invasion
BMI:	Body Mass Index
NLR:	Neutrophil-to-Lymphocyte Ratio
PLR:	Platelet-to-Lymphocyte Ratio
LMR:	Lymphocyte-to-Monocyte Ratio
pCR:	Pathological Complete Response
TLS:	Tertiary Lymphoid Structures
SRC:	Signet-Ring Cell
MSI:	Microsatellite Instability
PD-L1:	Programmed Death-Ligand 1
MIF:	Macrophage Migration Inhibitory Factor
TNF-α:	Tumor Necrosis Factor Alpha
IL-6:	Interleukin-6
IQR:	Interquartile Range
pTNM:	Pathological Tumor-Node-Metastasis
RFS:	Recurrence-Free Survival
NS:	not significant
